# Self-administered sexual health testing in an open prison setting: a pilot health impact assessment and social return on investment analysis

**DOI:** 10.1108/IJOPH-03-2024-0011

**Published:** 2024-11-12

**Authors:** Kathryn Ashton, Aimee Challenger, Christie Craddock, Timo Clemens, Jordan Williams, Oliver Kempton, Mariana Dyakova, Liz Green

**Affiliations:** Department of Care and Public Health Research, Maastricht University, Maastricht, The Netherlands and Department of Policy and International Health, Public Health Wales NHS Trust, Cardiff, UK; Department of Health Protection, Public Health Wales NHS Trust, Cardiff, UK; Department of Care and Public Health Research, Maastricht University, Maastricht, The Netherlands; Department of Policy and International Health, Public Health Wales NHS Trust, Cardiff, UK; Envoy Partnership, London, UK; Department of Policy and International Health, Public Health Wales NHS Trust, Cardiff, UK; Department of Policy and International Health, Public Health Wales NHS Trust, Cardiff, UK and Department of Care and Public Health Research, Maastricht University, Maastricht, The Netherlands

**Keywords:** Sexual health, HIA, SROI, Public health, Social value

## Abstract

**Purpose:**

The sexual health of the male prison population is often among the poorest in a country. This paper aims to identify the wider health impacts and social value of a sexual health self-sampling programme offered to male prisoners in an open prison setting in Wales.

**Design/methodology/approach:**

This study applied a unique pilot approach of using Health Impact Assessment and Social Return on Investment Frameworks in tandem. Key stakeholder groups affected by the intervention were identified, and engaged with through workshops, interviews and questionnaires to identify and quantify the health impacts and wider outcomes. Outcomes were then valued using proxy financial values to present the overall estimated social value of the self-sampling service.

**Findings:**

Based on a small sample, results indicate that for every £1 spent on the self-sampling service in the prison, a potential value of £4.14 was created. This resulted in a ratio of £4.14:£1. Approximately one-third of the value created (£1,517.95) was categorised as monetarily returnable, whereas the remaining value (£3,260.40) was purely illustrative social value, for example improved mental well-being.

**Originality/value:**

This unique pilot study demonstrates the health impacts and wider social value of providing a self-sampling sexual health service to prisoners within an open prison setting. By innovatively testing the feasibility of using a Health Impact Assessment process alongside Social Return on Investment analyses, this paper has outlined how the frameworks can be used in synergy to illustrate not just direct return on investment but also the social value of providing such a service.

## Introduction

The sexual health of men within the prison population is often among the poorest in any given country, as a result of poorer use of protection and engagement in casual sexual activities (Templeton *et al.*, 2019). This has resulted in a higher rate of transmission of sexual infections. Evidence suggests that infections such as chlamydia and gonorrhoea are less understood within the prison population compared to the wider community (Butler *et al.*, 2012). Chlamydia and gonorrhoea are sexually transmitted infections (STIs), that while being largely symptomless in many infected individuals, can cause significant adverse health outcomes if left untreated (NHS, 2021a, 2021b). For example, chronic pelvic pain, epididymitis and pelvic inflammatory disease (Li *et al.*, 2023). The identification and subsequent treatment of these infections is a key public health issue, and one which potentially has a number of other societal benefits, such as impact on sexual partners and potential impacts on mental well-being (Singh and Singh, 2021; Uzdavines *et al.*, 2022). In addition, prisoners could potentially return to the community with an infection, (particularly those in an open prison setting of a transient nature), which is an important public health issue (World Health Organization, 2014).

It is well documented that prisoners should be offered health care that is equivalent to the care provided in the community (RCGP, 2018; World Health Organization, 2014). This is important as it refers to elements of social justice and the reduction of health inequalities by ensuring individuals who are secured in environments such as prisons have equal access to service (World Health Organization, 2014).

**Figure 1:**
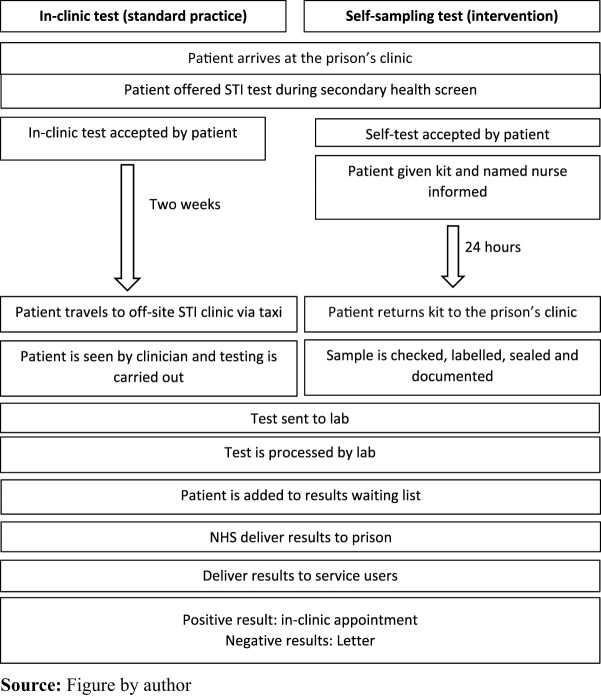
Standard practice versus self-sampling

As a result of the COVID-19 pandemic in Wales, individuals in the community were offered a service where samples could be taken independently, without the need to access a sexual health clinic (known as the Test and Post service (NHS Wales, 2023)). However, this service was not accessible by prisoners due to a lack of access to a phone or postal services. Due to this, an analogue version of the community test and post service in Wales was established within an open prison setting for male prisoners (i.e. prisoners can leave the setting for work or education purposes). Mirroring services available to the wider community, prisoners who presented to health-care workers with symptoms were given the opportunity to use a self-administered test as opposed to the in-clinic service traditionally offered by the prison setting (Figure 1). The self-administered tests include equipment to carry out triple site testing (urine, rectal and throat) in the privacy of their own cell. The prisoners then return their samples to the health-care team on site, who post them to the off-site sexual health clinic. Results are received in the same way as standard care.

The majority of existing evidence focuses on assessing the cost-effectiveness of STI testing, using health-care administered tests (Bagnall *et al.*, 2015; Castillo-Laborde *et al.*, 2021; Tuli and Kerndt, 2009). Although STI testing in prisons has previously been evaluated through an economic lens, to our knowledge, there is no existing evidence which looks to evaluate the economic case for self-administered testing within the prison setting.

There has been a growing demand for the public sector to develop methods for assessing how the use of public money can most effectively meet social, economic, and environmental needs and goals, maximising value (Ashton *et al.*, 2020b; Crown Commercial Service, 2023). The concept of value has shifted from purely an economic lens towards one that considers the wider impacts of an activity (Social Value UK, 2022). Measuring and capturing the wider impact of value of public health interventions is imperative to help make the case for investment in prevention, maximise limited resources and provide value for money while responding to growing health inequalities across communities and societies (Ashton *et al.*, 2022; Banke-Thomas *et al.*, 2015). This broader concept of value has been termed ‘social value’ (Banke-Thomas *et al.*, 2015; Social Value UK, 2012), which takes into account the economic, social and environmental benefits to an area, community or group of stakeholders. The Expert Panel on Effective Ways of Investing in Health [Directorate-General for Health and Food Safety (European Commission), 2019] link this to four value-pillars: allocative value (equitable distribution of resources), technical value (attaining the best possible outcomes), personal value (achieving patients’ individual goals) and societal value (including social participation). Previous studies have touched on the wider social value, including benefits to wider stakeholders, such as partners outside of prison (Dauria *et al.*, 2015; Nowotny *et al.*, 2020). However, these studies do not assign a monetary value to those wider social outcomes.

Building on previous research which highlights the synergies between Health Impact Assessment (HIA) (World Health Organization, 2023) and Social Return on Investment (SROI) (Ashton *et al.*, 2020a; Social Value UK, 2012), this paper explores how the two frameworks of HIA and SROI can be used to capture the health and equity impacts and economic value of the sexual health self-sampling programme during 2023. This study aims to explore and better understand the wider health impact and social value of the self-sampling service for the sexually transmitted infections of chlamydia and gonorrhoea, in an open prison setting. The results of this feasibility study can be used to demonstrate the wider impact and value of a self-sample service and can be used to advocate for its use across a wider range of prison settings if results indicate a positive impact and social value.

## Methods

HIA and SROI both capture health and well-being impacts and outcomes related to the wider determinants of health (Dahlgren and Whitehead, 2021). Both approaches follow clear processes and steps to capture a programme’s potential social, economic and environmental impacts and outcomes on health and well-being (Supplementary Tables 1 and 2). HIA as practised in Wales uses defined checklists for identifying the population groups and wider determinants of health which may be impacted by a programme, project or policy (Wales Health Impact Assessment Support Unit, 2012). In addition, SROI also considers the positive and negative effects of a policy or programme on the health of a population (Banke-Thomas *et al.*, 2015; Social Value UK, 2012). The SROI framework builds on HIA by using a health economics lens to quantify and value the wider impacts and outcomes identified as part of a HIA. The process carried out is described in Table 1.

### Stage 1: establishing scope and identification of stakeholder groups

A working group was established consisting of Public Health Wales representatives from the HIA support unit and sexual health and health protection services, prison and health-care service staff, and SROI experts. The working group used the HIA scoping checklist (Wales Health Impact Assessment Support Unit, 2020a) to set the parameters for the study and identify stakeholders who would potentially experience a change (whether positive or negative) due to the self-sampling intervention. The stakeholders identified were service users (prisoners accessing the health-care services), His Majesty’s Prison and Probation Services (HMPSS) and National Health Service Wales (NHS). Family members and sexual partners of the services users were also identified, but excluded from the analysis as the study team were unable to engage with these groups due to ethical constraints such as identifying and contacting individuals.

As per the NHS Research Authority decision-tool, NHS Research Ethics were deemed as not required for this project (Health Research Authority, 2022) as participants were not randomised to different groups, treatment/care/services were not changed from accepted standards and results from this pilot methodological study were not aimed to be generalisable. HMPPS National Research Committee (UK Government, 2024) reviewed and approved the project.

### Stage 2: Mapping outcomes

Representatives from each of the three stakeholder groups were invited to participate in primary qualitative research to identify the main outcomes experienced as a result of the self-sampling service. A HIA participatory workshop was held in December 2022 with representatives from HMPSS and the NHS, for example prison security staff and health-care clinicians. The workshop used the wider determinants of health and population groups checklists to define outcomes experienced by the different stakeholder groups (Wales Health Impact Assessment Support Unit, 2020b). An extra two semi-structured interviews were undertaken with representatives of the NHS and HMPSS who were unable to attend the workshop. In addition, semi-structured interviews were carried out with service users who had been identified by prison health-care staff as having used the self-sampling service. Prison staff approached services users to participate and informed consent was provided by the service user prior to the interview. All service users remained anonymous to the interviewers with no personal details or health-care records being accessed by the research team. Interviews were carried out both in person and virtually. Topics covered in the interviews were about their experience of the self-sampling service to identify outcomes. All interviews were recorded with the permission of the interviewee and anonymously transcribed. Notes from the interviews and workshop were analysed thematically by two members of the study team to allow for emerging themes to be mapped.

### Stage 3: valuing and evidencing outcomes

The outcomes for the service users were on a per-service user basis. As a result, service users were classified into different groups depending on:

- whether they would have done a sexual health test anyway if the self-sampling service was not available;
- whether their test was initially corrupted (i.e. neither a positive or negative results could be identified); and
- whether their test results were positive or negative.

In contrast, the outcomes for the HMPSS and NHS stakeholder groups were calculated on a per-test basis. This is because these stakeholders have to pay for resources on a test-by-test basis.

To enable the development of descriptions and indicators for each outcome, a quantitative survey was disseminated by health-care staff among all service users who attended the health-care prison service in June 2023. Service users were given the opportunity to answer questions about themselves, their sexual health, the sexual health services they had used at the prison and their future test preferences. The survey data was entered in Excel and analysed using basic statistical frequency tables. Each outcome was then valued using proxy financial values as per standard SROI methodology (Social Value UK, 2012).

### Stage 4: establishing impact

For all of the outcomes identified at previous stages, the proportion of change which was a direct result of the intervention was calculated. All variables outlined in Table 2 were accounted for.

All outcomes were given a value of zero with regards to displacement as they did not displace any other activities. All outcomes were given a score of 100% for attribution as all of the outcomes were caused as a direct result of the self-sampling intervention. Deadweight was accounted for by mapping the different routes service users could follow to obtain a test. As a result, it did not need to be accounted for in the impact calculation.

### Stage 5: the Social Return on Investment ratio

Using the proxy value, the value per year was calculated by multiplying the impact of the outcome, by the proxy value per stakeholder. Benefit period was also accounted for which takes into account how long the impact would have lasted for. The final value per outcome was then calculated and summed together to create the total value created by the self-sampling intervention. The SROI ratio was created by taking into account the total cost of running the intervention. A sensitivity analysis was also conducted to examine the influence of assumptions on the SROI model. Through an SROI process, assumptions are made such as assigning certain proxy valuations to the outcomes which do not hold a market of monetary value. The sensitivity analysis helps to account for this.

## Results

In total, four representatives from HMPSS and the NHS participated in the HIA workshop. In addition, one stakeholder and three service users participated in semi-structured interviews. This resulted in the identification of eight key outcomes that were included in the SROI analysis (Table 3). The additional stakeholder group of taxi drivers (who transport service users to the clinics) were also identified with an outcome of loss of income. However, this was excluded as it was assumed they would pick up different fares in substitution.

A total of 12 questionnaire responses were obtained from service users (the prison houses around 200 prisoners at one time), of which two respondents had used the self-sampling service. The age composition and employment status of survey respondents was comparable to the overall prison population (Supplementary Table 3). Due to the small response rate, it was recognised all results should be interpreted with caution and the analysis shifted to an assumption-based model based on data from both the questionnaire, but also existing prison data (Supplementary Table 4). This allowed for the number of stakeholders affected to be identified (Table 4), the change in outcomes per stakeholder to be calculated and subsequently the impact to be calculated per outcome (Table 5). Financial proxies were discussed within the research team to find the most suitable proxy using existing data form the literature or market values, and then applied to each outcome to allow for the total value to be calculated (Table 6). All financial proxies are designed to provide an indication of the value and should be used and interpreted with caution.

After taking into account the total cost of running the intervention over the study time period, i.e. the investment (£1,153.94, Supplementary Table 5), the overall potential total value of the intervention was calculated. It was calculated that for every £1 spent, the intervention created a value of £4.14. This resulted in a ratio of £4.14:£1. Approximately one third of the value created (£1,517.95) was categorised as monetarily returnable, whereas the remaining value (£3,260.40) was purely illustrative social value. The total value created for each stakeholder group was also calculated (Table 7).

Sensitivity analyses produced a range of SROI ratios from £3.22 to £5.46 for every £1 invested (Supplementary Table 6). The proportion of service users who would have completed the test anyway was the factor that produced the lowest overall SROI (£3.22:£1.00). A 50% reduction in the proportion of service users who would have completed an in-clinic test reduced the SROI by 22%. Workdays gained was the outcome that produced the lowest SROI. A 50% reduction in the attribution and financial proxy for workdays led to a 14% reduction in the SROI ratio (£3.58:£1.00). The number of stakeholders had the largest impact on the SROI ratio. A 50% reduction in the number of stakeholders increased the ratio by 32% to £5.46 per £1.00 invested, and it was predicted that a 50% reduction in the number of corrupted test would have increased the ratio by only 0.04% to £4.33 per £1.00 invested.

## Discussion

Although there have been previous economic evaluations of sexual health services within prisons (Bagnall *et al.*, 2015; Settumba *et al.*, 2018), this study pilots the use of an innovative methodology to analyse the impact and value of a self-sampling service through the lens of HIA and SROI. Using the HIA population groups and wider determinants checklists (Wales Health Impact Assessment Support Unit, 2020b), three main stakeholders groups were identified who have experienced change as a result of the intervention: service users, the NHS and the prison service. Each group experienced differing outcomes, which this study was able to quantify and value. This study has demonstrated how HIA can help an SROI analysis by directing it towards key stakeholders and population groups and focussing the conversation upon inequalities and vulnerable groups. Similarly, results show how SROI can assist HIA by monetising outcomes and help to build a more compelling case for investment in interventions that promote holistic health and well-being. Although previous research has indicated the direct return on investment of sexual health services in prisons (Gift *et al.*, 2006; Tuli and Kerndt, 2009), this study is unique in its contribution to the field of prison health research. By capturing the social value in addition to direct returns, results demonstrate the wider benefits of providing sexual health services in prisons on those wider determinants of health, as opposed to solely benefits to individual physical health.

Results show how it is feasible to provide a self-sampling service within an open prison setting. As prisoners are instantly provided with the swab kits to carry out the sampling themselves, the service falls well within NICE’s guidance of two days to expect to wait for a test (National Institute for Health and Care Excellence, 2019). It has also created a more equitable service for prisoners to access, mirroring services offered in the Welsh community (NHS Wales, 2023). It is also assumed that the burden placed on health board clinics is reduced due to the reduced need for appointments, particularly if this service was implemented in prisons with large populations. In addition to meeting national guidelines, this feasibility study shows that allowing service users to take their own samples for chlamydia and gonorrhoea within an open prison setting could potentially have generated £4,778.35 in social value for stakeholders. After this total value had been divided by the investment (or costs) of the intervention, the calculated SROI ratio was £4.14 for every £1 spent. This equated to £1.32 of tangible financial value being returned as a result of the investment for every £1 spent and £2.82 of illustrative wider social value being created as a result of every £1 spent. This illustrative value would not have been captured using traditional economic methodologies and reflects the value associated with improved mental health and well-being.

Within the study period, there were no positive infections of chlamydia or gonorrhoea identified within the prison setting. Despite this, a positive SROI ratio was reported, which can be attributable to reduced transport costs, a reduction in test waiting times and a reduced need to miss work or training due to attendance at external clinic appointments. However, it can be assumed that if any positive infections were identified, the value ratio would only increase due to the avoidance of negative impacts on physical health, as if left untreated, chlamydia can lead to pelvic inflammatory disease and further complications (Li *et al.*, 2023).

It is also important to consider that if the same analysis was to be undertaken in a closed prison setting, the value of the intervention would only increase. This is because prisoners have to be escorted off site to be taken to an external clinic. With the self-sample test, this cost would not exist so the savings to HMPPS would increase.

Finally, like in previous research (Ashton *et al.*, 2020a), the use of a combination of HIA and SROI to assess health impacts and social value, allowed for the well-rounded impact and value of the intervention to be demonstrated. Both processes consider the wider determinants of health and work well together to not only identify outcomes and impacts but also quantify and value them. The use of the HIA checklists provided structure to conversations and a clear and consistent process to follow in the workshops with participants. However, it was also noted, that neither HIA or SROI have a specific step or guidance on the development of a protocol. Although the scoping stages cover the main elements, a specific protocol would help guide transparency and additional detail around some of the methodological elements. In addition, clear communication was required to ensure the added value of running the two processes in combination was demonstrated to all involved in the study. Further reflections are outlined at Supplementary Table 7.

### Study limitations

This study is very much a pilot study which aimed to test the feasibility of using both HIA and SROI to assess the wider impact and value of an intervention. Because of this, and the small sample size who engaged in the research, results should be viewed with caution. Previous studies have highlighted that research involving prisoners is more difficult to carry out than research within a community setting (Sivakumar, 2021). The study team had limited access to the prison leading to limited options for stakeholder engagement, and a transient population in an open prison setting meant it was difficult to engage with a high number of service users within the study period. In addition, although sexual partners of the prisoners were identified as a key beneficiary due to earlier STI diagnosis and treatment, they were unable to be included within the scope of the analysis due to ethical constraints. Also, there was no baseline pre-intervention measure, elements of the study were based on assumptions and other relevant data sources. In addition, no randomisation or control group were used in the study and a number of elements of the SROI analysis was based on assumptions and all financial proxies have been chosen by the study team based on the best available data. However, this has been transparently reported throughout the paper and more information can be accessed from the comprehensive study report (Ashton *et al.*, 2024). Accurate uptake of the self-sampling test by service users as implementation of the intervention was not available from the prison health-care data, as only the number of tests given out was recorded. Data on corruption rates for in-clinic tests was also not available, so it was assumed the rate of corruption was the same for both the self-sampling and the in-clinic tests. Finally, the self-sampling programme was not advertised widely within the prison. Therefore, certain population groups may not have benefited from the campaign and the lack of advertising within the prison may impact the potential value generated by the intervention.

### Areas for future research

It is recognised this is an innovative feasibility study which promotes further opportunity to continue the development of analysis such as this. It would be interesting to understand the impact of the research and whether the monetization of impacts proved to be beneficial to stakeholders in showcasing the case for investment. All results should be viewed and interpreted with caution. It would be beneficial to carry out similar analyses to pilot the use of self-sampling testing interventions for other infections, such as blood-borne viruses within the prison setting, and in other types of prison settings such as closed prisons. In addition, due to the small sample size and feasibility nature of the study, it would be beneficial to carry out further research with the aim to obtaining a larger sample size to help provide assurance of validity to the results found in this study, and to critically appraise the added value, risks and benefits of this approach. Using both HIA and SROI frameworks in tandem can be built on going forward to develop a holistic framework to be used on other public health interventions to demonstrate not only impact on health and well-being but also on wider value. The use of the frameworks in tandem in other settings outside of the prison setting would develop this field of work further.

Finally, the process of valuing the outcomes in an SROI study such as this proved challenging without the use of a standardised proxy database. This is an area of research which should be prioritised if SROI is to be used consistently across studies to present accurate and valid valuations and findings.

## Conclusion

This study has not only highlighted the health and well-being impacts of the self-sampling sexual health service in an open prison setting but also demonstrated the social value of the service to the different stakeholder groups. Using an innovative approach of a HIA and SROI in tandem, this study has outlined the returnable and illustrative value of the intervention, through methods of stakeholder engagement, and assigning financial proxy values to a wide range of outcomes. This study provides a starting point for the future use of frameworks such as SROI not only in the field of prison health to effectively demonstrate the wider impact and value of interventions.

## Figures and Tables

**Table 1:** Analysis stages and how they map onto the stages of HIA and SROI frameworks

Stages of the study	How the stage maps to the HIA and SROI framework
Stage 1: Establishing scope and identification of stakeholder groups	HIA Stage 1: Screening to determine whether to complete a HIA HIA Stage 2: Scoping of the boundaries of the assessment SROI Stage 1: Establishing scope and identifying stakeholders
Stage 2: Mapping outcomes	HIA Stage 3: Evidence gathering and appraisal SROI Stage 2: Mapping outcomes
Stage 3: Valuing and evidencing outcomes	SROI Stage 3: Valuing and evidencing outcomes
Stage 4: Establishing impact	SROI Stage 4: Establishing impact
Stage 5: The SROI ratio	SROI Stage 5: Calculating the SROI
Stage 6: Reporting	HIA Stage 4: Reporting and recommendations SROI Stage 6: Reporting, using and embedding

**Source:** Table by authors

**Table 2:** Variable accounting for when establishing impact

Variable	Description
Deadweight	A measure of the amount of outcome that would have happened even if the activity had not taken place
Attribution	An assessment of how much of the outcome was caused by the contribution of other organisations or people
Displacement	When the benefits claimed are at the expense of others outside of the project

**Source:** Table by authors

**Table 3:** Key outcomes by stakeholder group

Stakeholder group	Outcome name
Service user (prisoner)	Workdays gained
	Education/training days gained
	Improved well-being (QALY)*
	Chlamydia: Improved physical health (QALYs gained)
	Gonorrhoea: Improved physical health (QALYs gained)
	Autonomy/value of the self-test
HMPPS	Reduced transport cost
NHS	Reduced sexual health clinic costs

**Note:** *QALY refers to “Quality Adjusted Life Years” which “measure the impact of disease on mortality into a single index” (Whitehead and Ali, 2010)

**Source:** Table by authors

**Table 4:** Number of tests taken per service user group

Total tests and service users during 1 year study period	Whether they would have completed the test or not dependent on service offered	Groupings	Test re-taken if corrupted
Self-sample tests completed: *n* = 54 Self-sample tests returned by service users: *n* = 40.60*	Servicer users who would have completed the in-clinic test anyway: *n* = 20.30	Group 1: test not corrupted *n* = 13.60	NA
		Group 2: test initially corrupted *n* = 6.70	And retaken *n* = 6.70
		Group 3: corrupted test not retaken *n* = 0	NA
	Service users who wouldn’t have completed the in-clinic test if the self-sample wasn’t available: *n* = 20.30	Group 4: corrupted test not retaken *n* = 0	NA
		Group 5: test negative *n* = 13.60	NA
		Group 6: test positive *n* = 0	
		Group 7: test initially corrupted *n* = 6.70	And retaken *n* = 6.70

**Notes:** *This is based on the fact some tests were corrupted, i.e. did not return a result and service users could have taken more than one test in the study period. The number of service users is not whole as we shifted to an assumption-based model, i.e. we knew the number of tests and the corruption rate so this is our estimate based on the number of service users

**Source:** Table by authors

**Table 5:** Total change per stakeholder (*s*-holder)

Outcome	S-holders affected	No. of potential *s*-holders	Indicator	Indicator %	Data source	No. of *s*-holders affected	Change per *s*-holder*	Total change per *s*-holder	Impact**
*Service users*									
Workdays gained	Group 1	13.601	% service users in employment	44	HMPPS	5.984	1	5.984	5.984
	Group 2	6.699				2.94756	2	5.895	5.985
Education/ training days gained	Group 1	13.601	% service users in education/ training	10	HMPPS	1.36	1	1.36	1.36
	Group 2	6.699				0.6699	2	1.3398	1.3398
Improved well-being (QALY)	Group 1	13.601	% with reduced waiting time	100	EQ-5D-5L	13.601	1	13.6	13.6
	Groups 5–7	20.3	% with reduced anxiety			20.3	1	20.3	20.3
Chlamydia: Improved physical health (QALY)***	Group 6	20.3	% who have a partner	42	Survey	8.526	0	0	0
Gonorrhoea: Improved physical health (QALY)***	Group 6	20.3	% who have a partner	42	Survey	8.526	0	0	0
Autonomy: value of self-test	Group 1	13.601	% who preferred self-test	62	Survey	8.432	1	8.432	8.432
	Group 2	6.699				4.153	2	8.306	8.306
*HMPPS*									
Reduced transport costs	All completed tests	54	% of transport costs saved	100	New versus old method	54	1	54	54
NHS									
Reduced sexual health clinic costs	All completed tests	54	% of clinic costs saved	87.5	New versus old method	47.248	1	47.248	47.248

**Notes:** *The change in outcome per stakeholder was calculated by subtracting the pre-intervention level of the outcome from the post-intervention level.

**after accounting for variables such as deadweight, attribution and displacement, as outlined in Methods section.

***only applicable to those individuals who have a partner as it was assumed only those who had a sexual partner could contract chlamydia or gonorrhoea for the purpose of this analysis

**Source:** Table by authors

**Table 6:** Valuing outcomes (*s*-holder = stakeholders)

Outcome	S-holders affected	Impact	Financial proxy per stakeholder: description	Financial proxy per *s*-holder: value	Benefit period: description	Benefit period: value	Drop off per year (%)	Value created per year
Workdays gained	Group 1	5.984	UK hourly minimum wage (£10.42) multiplied by a workday (7 h) (UK Government, 2024)	£72.94	1 day	1	100	£436.51*
	Group 2	5.895			2 days	2	100	£859.98*
Education/ training days gained	Group 1	1.36	Daily cost of bricklaying course (Total cost / Length of course = £2995 / 40) (Ableskills, 2023)	£74.88	1 day	1	100	£101.84*
	Group 2	1.3398			2 days	2	100	£200.65^*^
Improved well-being (QALY)**	Group 1	13.6	The smallest change on the EQ-5D-5L other than 0 (0.026) × NICE upper threshold (£30,000) (Appleby *et al.*, 2007; EQ-5D, 2023; McCabe *et al.*, 2008)	£780.00	13 days	0.0356	100	£377.85*
	Groups5–7	20.3					100	£563.95*
Chlamydia: improved physical health (QALY)****	Group 6	0	QALYs lost per 1 incident chlamydia infection (Li *et al.*, 2023)	£1,409.40	2 months	0.17	100	£0
Gonorrhoea: Improved physical health (QALY)****	Group 6	0	QALYs lost per 1 incident gonorrhoea infection (Li *et al.*, 2023)	£426.60	2 months	0.17	100	£0
Autonomy: value of self-test****	Group 1	8.432	Market value of a self-test for chlamydia and gonorrhoea (Superdrug, 2023)	£42.99	1 year	1	100	£362.52*
	Group 2	8.306					100	£357.11*
HMPPS								
Reduced transport costs	All completed tests	54	Saving made using new self-test method. Service users no longer require taxi rides to and from the off-site sexual health clinic	£20.00	1 year	1	100	£1,079.96
NHS								
Reduced sexual health clinic costs	All completed tests	47.248	Saving made using new self-test method. Service users no longer require a 20-min off-site	£9.27	1 year	1	100	£437.99
							total value of self-sampling	£4,778.35

**Notes:** *This was classified as an intangible cost, as the value would not be returnable to the stakeholder financially.

**It was not possible to collect (a) pre- and post-intervention data from the participants, or (b) representative data from the questionnaire. Therefore, to be conservative, we used the smallest possible change in anxiety on the EQ-5D-5L other than zero. This is the change from moderate anxiety (0.104) to slight anxiety (0.078). This resulted in a change of 0.026. Within the UK NHS, the effectiveness and cost effectiveness of treatments is evaluated by the National Institute for Health and Clinical Excellence (NICE) (32). At present, the NICE threshold currently ranges from £20,000 to £30,000 per quality adjusted life year (QALY) gained. However, the upper threshold of £30,000 has been chosen for this study as it is the method most frequently used by the NHS and the NHS is a stakeholder in this analysis. To calculate the QALY for anxiety/depression, the smallest amount of change (i.e. 0.104–0.078 = 0.026) was multiplied by the NICE upper threshold of £30,000;

***Data to calculate the chlamydia and Ggonorrhoea Improved physical health proxies was obtained from secondary research which expanded a probability-tree model to estimate the average number of life time QALYs lost due to genital chlamydia and gonorrhoea. To obtain the discounted lifetime QALYs lost per each infection, each of the figures was divided by 1,000. Thus, for each chlamydia infection, 0.04698 QALY were lost. This figure was multiplied by the NICE upper threshold to produce the financial proxy value per stakeholder (£1,409.40). Each gonorrhoea infection resulted in the loss of 0.01422 QALY. When multiplied by the NICE upper threshold, this produced a value of £426.60;

****This was based on how much an individual would be willing to pay to do an STI test in private

**Source:** Table by authors

**Table 7:** Total value created per stakeholder group

Stakeholder	Value created
Service users	£3,260.40
HMPPS	£1,079.96
NHS	£437.99

**Source:** Table by authors

## Supplementary Material

The supplementary material for this article can be found online.
